# Astrocytic Pathological Calcium Homeostasis and Impaired Vesicle Trafficking in Neurodegeneration

**DOI:** 10.3390/ijms18020358

**Published:** 2017-02-08

**Authors:** Nina Vardjan, Alexej Verkhratsky, Robert Zorec

**Affiliations:** 1 Laboratory of Cell Engineering, Celica BIOMEDICAL, 1000 Ljubljana, Slovenia; 2 Laboratory of Neuroendocrinology & Molecular Cell Physiology, Institute of Pathophysiology, Faculty of Medicine, University of Ljubljana, 1000 Ljubljana, Slovenia; 3 Faculty of Life Sciences, University of Manchester, Manchester M13 9PT, UK; 4 Achucarro Center for Neuroscience, IKERBASQUE, Basque Foundation for Science, 48011 Bilbao, Spain

**Keywords:** astrocyte, glia, vesicle traffic, calcium homeostasis, cAMP, neurodegeneration, locus coeruleus, noradrenaline, Alzheimer’s disease, neurodegeneration, Parkinson’s disease, excitation-energy coupling

## Abstract

Although the central nervous system (CNS) consists of highly heterogeneous populations of neurones and glial cells, clustered into diverse anatomical regions with specific functions, there are some conditions, including alertness, awareness and attention that require simultaneous, coordinated and spatially homogeneous activity within a large area of the brain. During such events, the brain, representing only about two percent of body mass, but consuming one fifth of body glucose at rest, needs additional energy to be produced. How simultaneous energy procurement in a relatively extended area of the brain takes place is poorly understood. This mechanism is likely to be impaired in neurodegeneration, for example in Alzheimer’s disease, the hallmark of which is brain hypometabolism. Astrocytes, the main neural cell type producing and storing glycogen, a form of energy in the brain, also hold the key to metabolic and homeostatic support in the central nervous system and are impaired in neurodegeneration, contributing to the slow decline of excitation-energy coupling in the brain. Many mechanisms are affected, including cell-to-cell signalling. An important question is how changes in cellular signalling, a process taking place in a rather short time domain, contribute to the neurodegeneration that develops over decades. In this review we focus initially on the slow dynamics of Alzheimer’s disease, and on the activity of locus coeruleus, a brainstem nucleus involved in arousal. Subsequently, we overview much faster processes of vesicle traffic and cytosolic calcium dynamics, both of which shape the signalling landscape of astrocyte-neurone communication in health and neurodegeneration.

## 1. Introduction

Locus coeruleus (LC) is a brainstem nucleus located in the pons in the lateral floor of the fourth ventricle, which contains neurones with neuromelanin granules. These give LC colouring and an alternative name of nucleus pigmentosus pontis or “heavily pigmented nucleus of the pons” [1]. This coloration results from the polymerization of noradrenaline (NA) and is analogous to the black dopamine-based neuromelanin. The LC thus is the primary source of NA in the central nervous system (CNS) [2,3,4]. When the LC neurones fire, this results in an activation of astrocytes in the cortex [5,6] and this likely fails to happen when LC is degenerated [2,7]. The LC, which contains a relatively small number of neurones (50,000 in adult humans [8]); the axons of these neurones project [3,9,10] to the brain stem, the spinal cord, the cerebellum, the hypothalamus, the hippocampus, the thalamic relay nuclei, the amygdala, the basal telencephalon, and the cortex along distinct bands [10]. These diffusely distributed nerve endings serve as an anatomical platform supporting synchronous and spatially homogeneous activation of neural networks in several regions of the brain and spinal cord [11]. This anatomical arrangement likely mediates the functional “reset” for many brain networks [12,13], as well as the most fundamental LC-mediated functions including arousal, attention, awareness, the sleep–wake cycle, memory formation, behavioural flexibility, behavioural inhibition and stress, cognitive control, emotions, neuroplasticity, posture, and balance [9].

LC nucleus is abundantly vascularised [14], indicating that LC neurones are metabolically demanding. They exhibit relatively high autonomous spiking rate even when glutamate and γ aminobutyric acid (GABA) transmission is blocked [15]. Reach vascularisation makes LC neurones exposed to circulating toxic substances, including viruses. Toxin accumulation in LC neurones may occur over long periods even when toxins are present at low concentrations and with limited blood–brain barrier penetration. Moreover, toxins could be taken up in sufficient quantities by LC terminal axons to be subsequently retrogradely transported to the cell body [16,17]. The LC proximal position to the fourth ventricle may further facilitate the exposure of LC neurones to toxins present in the cerebrospinal fluid (CSF) [18]. Therefore, LC nucleus is vulnerable to environmental conditions, which may lead over decades to the development of neurological and neurodegenerative disorders, including Alzheimer’s diseases (AD), Parkinson’s diseases (PD), and other diseases [2,17].

## 2. Locus Coeruleus Contributes to the Development and Plasticity of Neocortex

The mechanisms that facilitate or prevent neurodegeneration are poorly understood, however they may be related to the global coordinating function of LC that starts in prenatal period when the nucleus is already formed. It has been noted that LC efferents are associated with the development of various brain areas, especially the neocortex [3]. In rats, neurodevelopmental influences of LC neurones on neocortex start at 10–13 days of gestation [19], much earlier than neurones in the brain areas innervated by LC appear [3]. Using tyrosine hydroxylase (TH) immunocytochemistry, the LC neurones are detected, in humans, at 12 weeks of gestation, whereas they are absent at 5 weeks. At this age, labelled axons are also visible in catecholamine (CA) terminal areas. By 17–21 weeks of gestation, LC and other CA cell groups have coalesced into distinct cell clusters, and neuronal perikarya and processes have become more differentiated [20]. Morphological identification of LC nerve endings in the neocortex indicates that NA is involved in the development [21]. Axon terminals with NA are first located in the lower part of the cortical marginal zone, the site of tangential axons of Cajal-Retzius cells. These principal cells provide cues for the migration of cells born later in development and are involved in the neocortical lamination [22,23]. Early NA input appears to target Cajal-Retzius neurones [24], since removal of the NA system after birth resulted in an altered number of Cajal-Retzius cells [25].

Developing brain is growing in mass, which is associated with a problem for cell-to-cell signalling. Distances between cells become far greater than those reachable by diffusion–based signalling [26]. To bypass these obstacles, two mechanisms operate in the developing brain. The first is represented by convection-based signalling, where substances in the extracellular solution are transported by the bulk flow. This flow varies diurnally appearing to be the strongest during sleep, when the flux of CSF increases, thus helping to remove the extracellular debris [27]. Changes in the flux of CSF and the tortuosity of extracellular space are regulated by adrenergic receptors (ARs) [27,28], which also regulate astroglial morphologic plasticity [29,30]. Second mechanism for distant cell-to-cell signalling is represented by propagating action potentials. During development the propagation of action potentials along the ensembles of branching LC neurones may play a prominent role [3], because of their wide innervation of brain structures [9].

Activity of LC neurones affects the tortuosity of the extracellular space in the CNS, as well as volume and CSF flux acting primarily on astroglia. Several studies support this notion. An early electron microscopy report [31] demonstrated that there is a nine-fold greater number of β-ARs on astrocytic processes compared to neuronal processes in adult cortex, indicating that non-neuronal structures are more sensitive to NA. In support of this conclusion a recent study using brain slices and monitoring calcium (Ca^2+^) changes revealed robust NA-mediated signalling predominantly through α_1_-ARs in neocortical astrocytes but not in neurones [32]. Moreover, in vivo studies monitoring cytosolic Ca^2+^ activity in astroglia revealed that the primary target of NA appears to be a synchronous response in the most, if not in all of astrocytes in the microscope field [5,6]. Additionally, it is important to note that β-ARs are expressed on astrocytic processes [31], which may play a function in regulating the shape of astrocytes via cytosolic cAMP [29]. These astrocytic processes may get removed from the synapse upon memory formation [33].

The aforementioned action of NA released from LC neurones, which primarily affects astroglia [32], likely gets altered during neurodegeneration. In addition to the impaired morphological plasticity and signalling, which will be discussed below, it is relevant to note that astroglia is an important energy producing hub [34]. Developing tissues, where cells divide and undergo morphological plasticity, consume substantial energy [35]. Aerobic glycolysis, a non-oxidative metabolism of glucose which proceeds despite the presence of adequate levels of oxygen, known also as “the Warburg effect” [36] is an adaptation in growing tissues. While being inefficient to generate large amounts of ATP, this mode of metabolism provides intermediates for the biosynthesis of lipids, nucleic acids and amino acids [37]. Aerobic glycolysis is also associated with tissue plasticity in the adult CNS, especially in the frontal cortex [38]. Glycolytic intermediates are essential for biomass growth in cancer [35], indicating that cells exhibiting Warburg-effect are a universal feature of health and disease. The hallmark of aerobic glycolysis is the synthesis of L-lactate. Production of L-lactate and its release are up-regulated in the brain in states of alertness, attention, sensory stimulation, exercise, and in pathological conditions. Although the mechanisms are still unclear, it appears that at the cellular level, these processes likely depend on activation of astroglia by NA released from LC projections [11,39].

## 3. Atrophy of Locus Coeruleus and Neurodegeneration

A deficit in LC was initially proposed to be associated with the pathogenesis of idiopathic PD; subsequently views on neurodegeneration widened and PD is now viewed as a member of a family of diseases, which are neurological in nature, neurodegenerative in their progressive pathomorphological and functional characteristics and generally associated with ageing [2,4,17]. A sufficient plastic reserve appears to resist the ageing-associated deficits in LC [7]. However, the cellular mechanisms of the loss of LC neurones in neurodegeneration are unclear.

Perhaps the most compelling evidence of the role of the integrity of the LC in neurodegeneration comes from a longitudinal cohort study [7], where 165 patients were evaluated annually by 19 cognitive tests used for a composite measure of global cognition. After death, brain autopsy and neuropathological examination was performed to determine the density of neurones in the LC and in other brainstem nuclei. Measures of neuronal neurofibrillary tangles and Lewy bodies (likely due to the accumulation of α-synuclein [40]) from these nuclei as well as from medial temporal lobe and neocortex were obtained. The presence of pathological changes such as tangles and Lewy bodies in the brainstem nuclei were associated with accelerated cognitive decline [7]. This study confirmed the hypothesis that higher neuronal density in LC is a structural indicator of plastic reserve that limits the impact of neurodegenerative lesions on cognitive function. Therefore, a strategy of preserving the viability of neurones in LC and/or mimicking the action of NA in targeted areas is a valid and sensible strategy to mitigate neurodegeneration and age-dependent cognitive decline. This strategy also considers astroglial cells as signal integrators and energy providing entities in the CNS [34]. These functions, however, depend on morphological and signalling integrity of astrocytes. In both of these processes vesicle traffic is of particular importance.

## 4. Astrocytic Morphologic Dynamics and Neurodegeneration 

Neuronal networks are connected through (mostly) chemical synapses, many of which are enwrapped by astroglial processes, with the single astrocyte associating with many synapses. In the CA1 area of the adult rat hippocampal synaptic density is ~213 synapses/100 µm^3^ [41]. Since the estimated volume of a rat astrocyte is ~66,000 µm^3^, an individual astrocyte in rat hippocampus can be linked to ~140,000 synapses [42]. Human hippocampal astrocytes are larger and a single human astrocyte can cover up to ~2 million synapses [43]. The NA released from LC projections predominantly activates astroglia through α- and β-ARs, which are abundantly expressed in white and grey matter astrocytes [5,31,44,45,46]. Activation of ARs also instigates changes in cell morphology [47]. That is, stimulation of β-AR increases intracellular cAMP [29], which induces stellation of cultured astrocytes, i.e., transformation from a flattened irregular morphology to a star-like shape [29,48,49]. In vivo inhibition of β-ARs suppresses reactive gliosis [50,51], indicating the involvement of noradrenergic stimulation and cAMP signalling in the transformation of resting astrocytes into reactive ones. Morphological association between a synapse and a perisynaptic astroglial process can be dynamically modified during memory formation [33], that also requires NA and β-AR activation [52]. Morphological remodelling of astrocytes is also associated with pathological changes (e.g., astrocyte swelling during brain oedema formation). Recently, it was shown that NA, likely acting through β-ARs, attenuates acute cytotoxic oedema of astrocytes in response to hypotonicity and neurotrauma [30].

Morphological changes of astrocytes in neurodegeneration likewise occur at a slow time-scale. In neurodegenerative disorders this leads to a progressive loss of CNS function, due to a decrease in number and deterioration in structure of neural cells, ultimately resulting in the atrophy of the brain and in profound cognitive deficits. This is associated with aberrant protein synthesis reflected by accumulation of pathological proteins (such as β-amyloid or α-synuclein) either inside the cells or in the brain parenchyma; these alterations are accompanied with pathological changes in astroglia [53]. Signs of astroglial atrophy and astrogliotic activation have been observed at the presymptomatic phase of AD in humans even before the formation of β-amyloid deposits [54]. Occurrence of atrophic astrocytes appears to precede astrogliosis that develops in response to disease-specific lesions [55,56]. Similarly, in amyotrophic lateral sclerosis (ALS), astrodegeneration and astroglial atrophy occur before clinical symptoms and may be a key factor instigating neuronal death. In the animal model of ALS, expressing human mutant superoxide dismutase 1 (Tg(SOD1*G93A)1Gur mice), atrophic astrocytes appear to be the earliest pathological signature [57,58]. These atrophic astrocytes have a reduced ability to remove glutamate, hence extracellular glutamate accumulates with ensuing excitotoxicity [59]. At the later stages of ALS, a sub-populations of astrocytes also becomes reactive, albeit atrophic forms remain. The importance of astrocytes in ALS pathogenesis is further corroborated by the observation that silencing the ALS-related mutant SOD1 gene specifically in astrocytes delayed the appearance of disease symptoms in the transgenic mouse model [60]. Astroglial degeneration with loss of function characterized by a significant down-regulation of astroglial glutamate transporters resulting in prominent excitotoxicity is manifest in Wernicke encephalopathy, a thalamo-cortical neurodegeneration that represents the morphological substrate of Korsakoff syndrome [61,62]. Similarly, in Huntington disease (HD), a decreased astroglial glutamate uptake as well as an aberrant release of glutamate from astrocytes contributes to neurotoxicity [63]. Suppression of astrogliotic response by inhibition of JAK/STAT3 signalling cascade increases the number of huntingtin aggregates [64], thus exacerbating pathological progression. In the context of PD, astrocytes are supposed to play a neuroprotective role [65,66]. Astrocytes were also shown to convert l-3,4-dihydroxyphenylalanine (l-DOPA) to dopamine [67]. In the striatum, astrocytes act as a reservoir for L-DOPA, which they release to be subsequently transported to neurones [68]. Expression of glial fibrillary acidic protein (GFAP) was decreased in astrocytes in PD human tissue [69], indicating astroglial atrophy and reduced astrogliotic response, which may reflect compromised astroglial neuroprotection.

## 5. Vesicle Traffic, Surface Signalling Landscape of Astrocytes, and Neurodegeneration 

Vesicle traffic, with a dynamics in minutes, contributes to morphological plasticity of all cells, also in pathology, since membrane added to the plasmalemma during exocytosis and then taken away by endocytosis may change drastically with disease. The consequences of imbalanced exo- and endocytotic vesicle traffic alters cell surface signalling landscape of astrocytes [70]. Vesicles, carrying signalling molecules in their lumen as well as receptors and transporters in their membrane, usually originate in the secretory pathway, from the Golgi complex, deep in the cytoplasm, and are then trafficked to the cell surface. Once the vesicle membrane merges completely with the plasmalemma, this vesicle incorporated membrane may, at a later stage, give birth to an endocytotic vesicle which can travel into the cytoplasm or may recycle back to the plasma membrane.

In astrocytes vesicle traffic is maintained by an elaborated system regulated by fluctuations in [Ca^2+^]_i_ [70,71]. The complexity of vesicle traffic regulation in astrocytes is characterized by two typical, yet opposing, properties of vesicles that contain peptides, such as atrial natriuretic peptide (ANP), and those that carry amino acid transmitters and are labelled by the vesicular glutamate transporter VGLUT1 [70,71,72]. Glutamatergic vesicle mobility is accelerated by an increase in [Ca^2+^]_i_ [73], whereas the same increase in [Ca^2+^]_i_ slows down peptidergic vesicles and endolysosomes [74]. Similar regulation also applies to recycling peptidergic vesicles, which have merged with the plasma membrane and subsequently entered back into the cytoplasm. The mobility of recycling peptidergic vesicles was studied in astrocytes in culture [75] and in the brain slices [76]. In these two preparations, at rest, peptidergic vesicles moved faster and more directionally, being linked to cytoskeletal elements [75]. The effect of increased [Ca^2+^]_i_ was remarkable: the movement of vesicles was almost halted, with only a jitter remaining (that was associated with random diffusional movement). At least some of the peptidergic vesicles carry ATP and a similar attenuation of their mobility was observed when astrocytes were stimulated [77].

When vesicle mobility is suppressed, their increased residency at the plasma membrane may increase probability that the vesicle membrane interacts with the plasma membrane. This is facilitated by the *n*-ethylmaleimide-sensitive fusion factor attachment protein receptor (SNARE) complex formation [78]. By using a dominant-negative domain of synaptobrevin 2 protein (dnSNARE), a vesicle-based SNARE protein, it was shown, at the level of single astrocytic vesicle, that disassembly of the SNARE complex mediates the full merger between the vesicle and the plasma membranes [79]. In other words, when a vesicle interacts with the plasma membrane, for which SNARE complex formation is needed, and when the SNARE complex cannot be disassembled, fusion pore maintains in open and stable configuration while the vesicle membrane is unable to merge completely with the plasmalemma. This process may be regulated by endogenous lipids, such as sphingosine, which can be generated on the outer leaflet of the plasmalemma, followed by subsequent internalization across the plasma membrane into the cell [80,81]. Sphingosine is considered to facilitate regulated exocytosis by recruiting vesicle synaptobrevin 2 for the SNARE complex formation [82]. It appeared that sphingosine modulates the formation of the fusion pore, the latter being a channel that forms between the vesicle and the plasma membrane, a process that is also strongly influenced by vesicle size in endocrine cells [83] and in astrocytes [79]. In astrocytes, sphingosine analogue FTY720 (fingolimod), currently used for treatment of multiple sclerosis, strongly affects vesicle mobility and the release of peptides from a single vesicle [84]. This is associated with a change in Ca^2+^ homeostasis in astrocytes [85].

The process of vesicle interaction with the plasma membrane is also a target of ketamine, an anaesthetic and an antidepressant. Unlike classical antidepressants, ketamine demonstrates both fast and sustained effects [86], indicative of a fundamentally different mechanism of action that may alter synaptic efficacy [87]. Ketamine affects astrocytes by inhibiting the release of brain derived neurotrophic factor BDNF [88]. Membrane capacitance measurements (used to monitor the interaction of a single astrocytic vesicle membrane with the plasmalemma) revealed that sub-anaesthetic doses of ketamine stabilize the fusion pore in a narrow flickering state [89], the latter being too narrow to allow the discharge of vesicle content into the extracellular medium [88]. Therefore, vesicle traffic and the fusion pore can be a target for therapy, possibly not only for treating depression and neurodegeneration in multiple sclerosis, but also for other neurological conditions.

In neurodegeneration, as in AD, a vesicular traffic deficit could be a primary mechanism of the early, pre-symptomatic stage of the disease, possibly associated with alterations in the signalling profile of astrocytes [70] and the removal of β-amyloid from the extracellular space [90]. For example, endolysosomes store and release proteolytic enzymes, such as insulin degrading enzyme (IDE), one of the major proteases of β-amyloid peptide, which may contribute to the development of AD. When secreted to the extracellular space IDE may degrade β-amyloid. While IDE is secreted primarily from neurones in the healthy brain [91], in AD astrocytes become the major cell type secreting IDE [90,92]. It has been proposed that in AD the capacity of astrocytes to secrete IDE is reduced when compared to healthy astrocytes. This leads to an increase in β-amyloid accumulation, which involves a reduction in autophagy-based lysosomal secretion of IDE [90]. Why this reduction occurs is not clear, but it may relate to a general vesicle traffic impairment that has been observed in AD and may involve lysosomal capacity to repair injured astrocytes [93].

**Figure 1:**
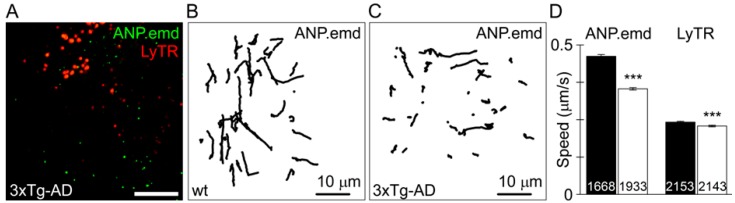
Diminished mobility of peptidergic (ANP.emd) and acidic vesicles in astrocytes from an animal model of Alzheimer’s disease (3xTg-AD). (**A**) A double fluorescent confocal image of the 3xTg-AD astrocyte expressing ANP.emd stored in individual vesicles observed as bright green fluorescent puncta and LysoTracker-labeled (LyTR) vesicles observed as red fluorescent puncta; scale bar, 10 µm; (**B**) Vesicle tracks (*n* = 45) obtained in a 15-s epoch of imaging representative control (wt); and (**C**) 3xTg-AD astrocytes expressing ANP.emd. Note less elongated vesicle tracks in the 3xTg-AD astrocyte. (**D**) Speed of ANP-loaded vesicles and LyTR-labelled vesicles in wt (black bars; mean ± SEM) and 3xTg-AD astrocytes (white bars). Note substantially diminished speed of peptidergic vesicles and modestly diminished speed of LyTR-labeled vesicles in 3xTg-AD astrocytes. The numbers at the bottom of the bars indicate the number of vesicles analyzed. *** *p* < 0.001 versus wt (Mann-Whitney *U* test). Modified with permission [94].

Astrocytes from 3xTg-AD mice isolated in the pre-symptomatic phase of the disease exhibit alterations in vesicle traffic. Spontaneous motility of peptidergic and endolysosomal vesicles as well as the ATP-evoked, Ca^2+^-dependent, vesicle mobility are all diminished in diseased astrocytes (Figure 1). Similar impairment of peptidergic vesicle trafficking was observed in healthy rat astrocytes transfected with familial AD-associated mutated presenilin 1 (PS1M146V). The stimulation-dependent peptide discharge from single vesicles was less efficient in 3xTg-AD and PS1M146V-expressing astrocytes than in respective controls. The impaired vesicle dynamics and reduced evoked secretion of the signalling peptides may contribute to the development of AD [94]. Although in this study ANP-containing vesicles were examined, it is likely that all peptidergic vesicles exhibit similar changes. Moreover, peptidergic secretion appears to be impaired also in HD by mutated huntingtin [95].

## 6. Calcium and cAMP Signalling in Astrocytes

Unlike neurones, which exhibit electrical excitability (firing action potentials generated at the level of the plasmalemma) that triggers neurotransmitter release from synaptic terminals, astrocytes are electrically silent and display intracellular excitability. The hallmark of cytosolic excitability is an increase in cytosolic levels of second messengers such as cyclic adenosine monophosphate (cAMP), Ca^2+^ and sodium (Na^+^) [96,97]. The overall evidence for Ca^2+^ as a second messengers in astrocytes exceeds that for cAMP, because Ca^2+^ recordings have been possible since 1990 [98], whereas real-time measurements of cAMP emerged only recently with the development of genetically encoded cAMP nanosensors [29]. It is generally acknowledged that changes in cytosolic Ca^2+^ follow a more phasic pattern, a consequence of the complex activation of various receptors, pumps and transporters; whereas cAMP levels change more tonically, following activation of cAMP-synthesizing enzyme adenylate cyclase [96,99].

**Figure 2:**
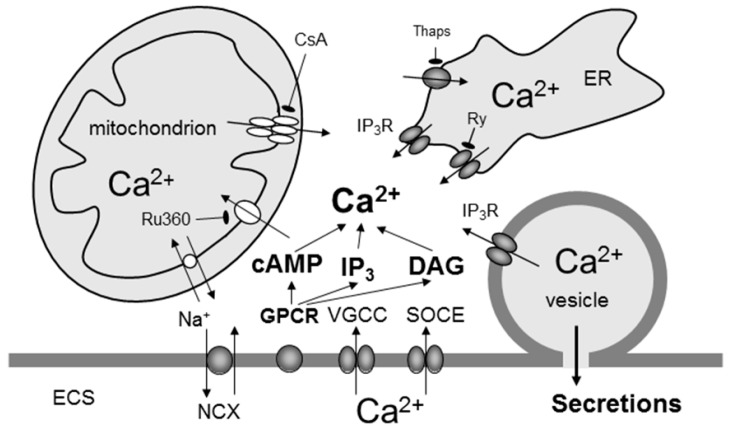
Interactions between Ca^2+^ and cAMP signalling for regulated vesicle-based secretion from astrocytes. The accumulation of Ca^2+^ in the cytosol may occur (1) following the entry of Ca^2+^ from the extracellular space (ECS) through L-type voltage-gated channels (VGCC), store-operated Ca^2+^ entry (SOCE) via transient receptor potential canonical type 1-containing channels, and the plasma membrane Na^+^/Ca^2+^ exchanger (NCX), and (2) via G protein-coupled receptor (GPCR) activation, which can generate the additional second messengers cAMP, inositol 1,4,5 triphosphate (IP_3_), and diacylglycerol (DAG). T bar denotes inhibition, arrows Ca^2+^ flux direction and interactions between second messengers. The GPCR activation in astrocytes retrieves Ca^2+^ from the endoplasmic reticulum (ER) internal stores that possess IP_3_ receptors (IP_3_R) as well as from ryanodine (Ry)-sensitive channels acting as conduits for Ca^2+^ delivery to the cytosol. The ER store is (re)filled by Ca^2+^-ATPase (i.e., SERCA pumps), which can be blocked by thapsigargin (Thaps). Cytosolic Ca^2+^ levels are modulated by mitochondria. These organelles take up Ca^2+^ via the Ca^2+^ uniporter, which is blocked by ruthenium 360 (Ru360), during the cytosolic Ca^2+^ increase. As cytosolic Ca^2+^ decreases due to the extrusion mechanisms, Ca^2+^ is slowly released by mitochondria into the cytosol via the mitochondrial Na^+^/Ca^2+^ exchanger as well as by the transient opening of the mitochondrial permeability transition pore. This transient opening is indirectly blocked by cyclosporin A (CsA), which binds cyclophilin D (not shown). The increase in cytosolic Ca^2+^ levels is sufficient and necessary to cause the fusion of secretory vesicles (which themselves can act as IP_3_-sensitive stores for Ca^2+^) with the plasma membrane, mediating the exit of gliosignalling molecules (such as amino acids, peptides, and ATP) from the vesicle lumen into the ECS. The cAMP-mediated modulation of Ca^2+^ homeostasis may occur at the level of Ca^2+^ entry or extrusion from the cytosol. Moreover, cAMP-mediated mechanisms may directly affect the fusion pore and the extrusion of gliosignals from the vesicle lumen. Drawing is not to scale. Reproduced with permission [104].

Astrocytes as neuronal partners in the multipartite synapse use various receptors to sense neurotransmitters released during synaptic activity, but they also detect a host of other signalling molecules present in the brain parenchyma, being for example transported with the convective flow of CSF, which is a part of the glymphatic system [100]. Many of these molecules are released by astrocytes themselves [101]. Binding of signalling molecules to their receptors affects not only [Ca^2+^]_i_ but also other cytosolic second messengers such as cAMP (Figure 2). For instance, binding of NA to astrocytic ARs triggers simultaneous, although temporally distinct, elevation of both second messengers in astrocytes. Such receptor mediated cytosolic excitability may lead to astroglial secretion of gliosignalling molecules, which in turn can interact with the receptors on synaptic terminals modulating neuronal excitability [102,103] or affect receptors on other neighbouring cells in a paracrine or autocrine manner [96,101].

Many of astroglial membrane receptors are high affinity metabotropic G protein-coupled receptors (GPCRs) [102,105,106]. Extracellular ligands activate GPCRs, which elicit a conformational change transmitting the signal to an attached intracellular heterotrimeric G protein complex. G protein subunit isoforms trigger distinct signalling pathways. For example, G_q_ subunit activation results in the stimulation of phospholipase C, which increases the concentration of diacylglycerol and inositol triphosphate (InsP_3_). In astrocytes, binding of InsP_3_ to InsP_3_Rs located on the endoplasmic reticulum (ER) [107] or on secretory vesicles [108] increases [Ca^2+^]_i_ through the release of Ca^2+^ from these organelles. Activation of ryanodine receptors on the ER may also increase cytosolic Ca^2+^ levels through Ca^2+^-induced release of Ca^2+^ from the ER [107]. The Ca^2+^ signal arising from the generation of InsP_3_ may be amplified by activating further Ca^2+^ release from InsP_3_Rs and ryanodine receptors [109]. In addition, mitochondria, otherwise acting as metabolic furnaces, have a role in Ca^2+^ buffering in astrocytes through Ca^2+^ uptake and release [110,111]. The Ca^2+^ can also enter astrocytes from the extracellular space through plasmalemmal Ca^2+^ channels [112,113,114], ionotropic receptors [115], and the Na^+^/Ca^2+^ exchanger [116] as well as through the transient receptor potential canonical type 1-channel [117] acting as a substrate for store-operated Ca^2+^ entry that, along with store-specific Ca^2+^-ATPase, replenish the depleted ER store. Although the mechanisms that can contribute to the elevation in cytosolic Ca^2+^ are many, it is universally accepted that the main pathway for global Ca^2+^ signalling is represented by GPCR activation and release of Ca^2+^ from InsP_3_-sensitive internal stores. Unlike G_q_ GPCR activation, stimulation of G_s_ GPCR subunits in astrocytes triggers adenylyl cyclase to catalyse the conversion of ATP to cAMP [29,118]. This cAMP activates intracellular effectors, primarily cAMP-dependent protein kinase A, which, by phosphorylating cytoplasmic and nuclear targets, mediates functional responses. Signalling via cAMP-activated GTP-exchange protein [119], cAMP-gated ion channels, and Popeye domain-containing proteins [120] may also contribute [121].

In astrocytes, G_q_-induced cytosolic Ca^2+^ increases occur as either oscillations or sustained elevations [102,105,122]. Astroglial Ca^2+^ excitability has been observed in culture [98], in brain slices in situ [123], and in vivo [124] and may occur spontaneously or in response to neuroligands, including neurotransmitters [98]. Ca^2+^ excitability can propagate from an excited astrocyte to its neighbouring unstimulated astrocytes in the form of intercellular Ca^2+^ waves, which are carried by diffusion of InsP_3_ through gap junctions [125] or via astrocytic release of ATP and subsequent receptor-mediated activation of adjacent astrocytes [126]. These waves travel at 10–20 μm/s [109]. In contrast, G_s_ activation induces persistent cAMP elevations [29,96]. It is currently unclear whether G_s_-induced cAMP excitability can be propagated in astroglial syncytium, as was observed for Ca^2+^ excitability. The G_q_- and G_s_-mediated pathways in astrocytes may interact, as activation of the G_s_-signalling pathway may potentiate G_q_-mediated Ca^2+^ responses [127] and vice versa [128,129].

Both G_q_ and G_s_ GPCR signalling pathways were shown to be involved in exocytotic release of chemical messengers from astrocytes. It is well established that Ca^2+^ elevation in astrocytes triggers the exocytotic release of glutamate [130,131,132,133,134], ATP [135,136], secretogranin II [103], ANP [137], and d-serine [138]. The role of cAMP in vesicle-based release of gliosignalling molecules from astrocytes is much less studied. Limited results indicate that increases in cAMP can trigger the discharge of secretogranin II from peptidergic vesicles [103]. In agreement with this, augmented Ca^2+^-triggered release of ANP was measured under conditions when membrane permeable cAMP analogue dibutyryl cAMP was introduced prior to stimulating astrocytes [139]. The mechanism of cAMP modulation of fusion machinery is not known. It may affect the discharge of gliosignalling vesicles de novo. It may also modulate the fusion pore dynamics as described in neuroendocrine cells [140].

Activation of Ca^2+^ and cAMP is involved, among others, in the regulation of energy needed for the morphological plasticity in the CNS. Although synapses are the main energy consumers in the brain, glycogen, the only CNS energy storage system, is present mainly, if not exclusively, in astrocytes. Memory consolidation associated with morphological plasticity of neurones and astrocytes requires (in young chickens) glycogenolysis [141,142]. The release of NA is required for memory consolidation [143]. Since NA, released from noradrenergic LC neurones, stimulates relatively rapid changes in astrocyte morphology following changes in Ca^2+^ and cAMP, this indicates that astrocytes act as signal integrators; they coordinate morphological and metabolic functions [144]. It was reported that NA is the main neurotransmitter that triggers astroglial Ca^2+^ signalling in the adult awake brain [5]. In addition it may trigger astroglial cAMP signalling via β-AR activation. Thus, astrocytes are hubs of NA mediated excitation-energy coupling in the CNS [144].

## 7. Altered Astroglial Calcium Homeostasis in Alzheimer’s Disease

Given that knowledge about Ca^2+^ signalling is far greater than that about cAMP signalling, it is logical that current views about alterations of astrocyte excitability focus mainly into Ca^2+^ homeostasis. In human astrocytes, isolated from the post-mortem temporal cortex obtained from three groups with different degrees of pathology in PD and AD (different Braak scores) 32 genes associated with Ca^2+^ signalling and homeostasis were identified to be abnormally expressed [145].

When astrocytes are exposed to β-amyloid, complex effects on intracellular Ca^2+^ signalling are observed [146,147] which may increase glutamate release from human astrocytes [148]. Resting [Ca^2+^]_i_ was increased several fold in astroglial cultures treated, for several hours, with oligomeric β-amyloid_1–42_ [149,150]. This was, however, not confirmed, when longer treatment times were used (e.g., 48 h exposure) and other amyloid peptides such as β-amyloid_1–40_ [151] or β-amyloid_25–35_ [152] were employed. In dissociated and organotypic brain cultures astrocytes may respond to β-amyloid treatment with the acute intracellular Ca^2+^ elevations, sometimes Ca^2+^ oscillations [153,154,155,156,157]. This was however not confirmed in some other studies [150,151,152]. The observed differences may be due to different species and concentrations of β-amyloid used (higher concentrations trigger Ca^2+^ elevations more readily). It has been suggested that β-amyloid can generally increase astroglial Ca^2+^ excitability [146,147] by increasing intracellular Ca^2+^ release and store-operated Ca^2+^ entry, SOCE [158,159].

An increased astroglial Ca^2+^ signalling has been reported in several AD animal models. In APP/PS1 mice [160] harbouring the mutant human β-amyloid precursor protein (APP_swe_) and mutant presenilin 1 (PS1ΔE9) an increase in the resting Ca^2+^ concentrations and Ca^2+^ hyperactivity with abnormal long-projecting intercellular Ca^2+^ waves were detected in astrocytes surrounding senile plaques. Similar high-frequency intercellular Ca^2+^ waves were detected in astrocytes at the pre-plaque stages in APP_Swe_ mice model [161]. The underlying cause of increased Ca^2+^ excitability of astrocytes from AD model mice may be an elevated ATP release from reactive astrocytes that leads to over-activation of P2Y_1_ purinergic receptors [162].

Abnormal Ca^2+^ signalling could be associated with the mutated presenilin 1 as observed in primary astroglial cultures from neonatal 3xTg-AD mice [150,159]. In cultured hippocampal astrocytes from 3xTg-AD mice exposure to ATP triggered larger Ca^2+^ oscillations compared to the control cells [163]. The SOCE was also increased in cultured 3xTg-AD astrocytes [159]. Moreover, expression of mutant presenilin 1 (M146V) impaired vesicular trafficking and secretion [94]. The above mentioned abnormalities are part of early pathological remodelling of astroglia and may be involved in development of AD pathology. In addition, deletion of amyloid precursor protein inhibited astroglial SOCE, likely via down-regulation of TRPC1 and Orai 1 Ca^2+^ channel expression [164]. On the contrary the over-expression of APP did not affect Ca^2+^ transients and SOCE in primary cultured astroglial cells from Tg5469 AD mice [164]. Resting Ca^2+^ levels were elevated two-fold compared to the controls also in astrocytes obtained from a Down syndrome mouse model (Trisomy 16 mice) that shares several key features with AD [165]. When these astrocytes were exposed to a sarco-endoplasmic reticulum Ca^2+^ transport ATPase (SERCA) inhibitor cyclopiazonic acid, which unmasks leakage of Ca^2+^ from the ER, large intracellular Ca^2+^ elevations were observed, reflecting higher ER Ca^2+^ content compared to controls. A positive correlation between the amplitude of cyclopiazonic acid-induced intracellular Ca^2+^ elevations and the resting Ca^2+^ levels was observed [165].

Chronic long-term exposure to β-amyloid changes the expression of ionotropic and metabotropic receptors, Ca^2+^-dependent enzymes, intracellular Ca^2+^ channels and SOCE. Upregulation of metabotropic glutamate receptor mGluR5 has been observed in vitro upon 24–72 h exposure of cultured astrocytes to oligomeric β-amyloid (100 nM to 20 μM) [150,151,163] and also in the brains of patients with Down’s syndrome [166], in cortical astrocytes found in the APP_swe_/PS1ΔE9 mice senile plaques [167], in post-mortem hippocampi of Braak V–VI stage AD patients [150] and late-stage sporadic AD cases [151]. Chronic exposure to low concentrations of β-amyloid_1–42_ (0.1–100 nM; [168]) increased expression of Ca^2+^ permeable α7 nicotinic cholinoreceptors in cultured astrocytes. This receptor has been found post-mortem in astrocytes of sporadic AD patients and in patients carrying the Swedish β-amyloid precursor protein mutation [169]. Another possible mechanism for observed abnormalities in astroglial Ca^2+^ signalling in AD pathology could result from direct β-amyloid activation of metabotropic receptors [170,171].

## 8. Conclusions

There are conditions where relatively large areas of the CNS need to be synchronously activated. This involves the stimulation of LC neurones and NA release, which primarily excites astrocytes, since the density of adrenergic receptors is almost an order of magnitude higher in astroglia vs. neurones [31,32]. Thus, primarily astrocytes respond to noradrenergic activation with elevations of intracellular Ca^2+^ and cAMP. Increased Ca^2+^ and cAMP excitability that occurs at short time-scale regulates astrocyte cellular metabolism, morphology and vesicle traffic. The latter controls the surface signalling properties of astrocytes and their capacity to provide sufficient amounts of energy via augmented aerobic glycolysis. These processes may be altered during decades in neurodegeneration, such as taking place in AD. If LC neurones, which are vulnerable due to their relatively high metabolic rate, are reduced in numbers, e.g., during neurodegeneration, NA-mediated synchronous brain “reset” is impaired. On the other hand, even if the LC neurones are intact, alterations in vesicle traffic of astrocytes, affecting signalling landscape of these cells, together with impaired astroglial Ca^2+^ and possibly cAMP systems may also play an additional role in facilitating the long-term course of neurodegeneration. Since the excitation-energy coupling occurs mainly in the astrocytes, more emphasis is needed to understand how astrocytes interact with the noradrenergic signals at the level of both Ca^2+^ and cAMP signalling, and how they cope with the demand of coherent spatio-temporal signalling in the relatively large brain areas taking place in a long-time domain.

## Abbreviations

CNS	central nervous system
CSF	cerebrospinal fluid
MS	multiple sclerosis
AD	Alzheimer’s disease
PD	Parkinson’s disease
HD	Huntington disease
ALS	amyotrophic lateral sclerosis
PKA	protein kinase A
PKC	protein kinase C
IP_3_	inositol triphosphate
GFAP	glial fibrillary acidic protein
SNARE	soluble *n*-ethylmaleimide-sensitive fusion factor attachment protein receptor
cAMP	adenosine monophosphate
ER	endoplasmic reticulum
ANP	atrial natriuretic peptide
LC	locus coeruleus
GPCR	G protein-coupled receptors
SOCE	store-operated Ca^2+^ entry
